# Ethnicity and the prostate cancer experience: a qualitative metasynthesis

**DOI:** 10.1002/pon.4222

**Published:** 2016-08-23

**Authors:** Carol Rivas, Lauren Matheson, Johana Nayoan, Adam Glaser, Anna Gavin, Penny Wright, Richard Wagland, Eila Watson

**Affiliations:** ^1^ Faculty of Health SciencesUniversity of SouthamptonSouthamptonUK; ^2^ Faculty of Health and Life SciencesOxford Brookes UniversityOxfordUK; ^3^ School of Medicine, Dentistry and Biomedical Sciences, Centre for Public HealthQueen's University BelfastBelfastUK; ^4^ Leeds Institute of Cancer and Pathology, Faculty of Medicine and HealthUniversity of LeedsLeedsUK

**Keywords:** ethnicity, oncology, patient experience, prostate cancer, qualitative metasynthesis

## Abstract

**Objectives:**

To summarize black and minority ethnic (BME) patients' and partners experiences of prostate cancer by examining the findings of existing qualitative studies.

**Methods:**

We undertook a systematic metasynthesis of qualitative studies using a modified version of Noblit and Hare's “meta‐ethnography” approach, with a 2000‐2015 search of 7 databases.

**Results:**

Thirteen studies of men from US and UK BME groups were included. We explored constructs with BME‐specific features. Health care provider relationships, formation of a spiritual alliance with God (which enhanced the participants' feeling of empowerment and ability to cope with the cancer), and living on for others (generally to increase cancer awareness), often connected to spiritual regrowth, were the 3 constructs most commonly reported. A magnified effect from erectile dysfunction was also common. Initially, this affected men's disclosure to others about their cancer and their sexual problems, but eventually men responded by shifting their conceptualizations of masculinity to sustain self and social identities. There was also evidence of inequality resulting from financial constraints and adversity that necessitated resilience in coping.

**Conclusions:**

The prostate cancer experience of BME men and their partners is affected by a complex intersection of ethnicity with other factors. Health care services should acknowledge this. If providers recognize the men's felt masculinities, social identities, and spiritual beliefs and their shifting nature, services could be improved, with community as well as individual benefits. More studies are needed in diverse ethnic groups.

## 1 Background

Prostate cancer (PCa) is the commonest cancer in men in developed countries^1^ and fourth most common worldwide, with over 1 million men diagnosed annually.^2^ However, there is a greater than 25‐fold variation in its regional annual incidence, with the highest in Northern and Western Europe, North America, and Australasia and the lowest in South‐Central Asia.^3^ Localized PCa may be treated with curative intent and even when metastasized frequently responds to treatment,^1^ but mortality rates and survivorship, like incidence, vary considerably by country.^4^ Critically, many countries do not have the resources for advanced, individualized PCa screening and care,^4^ explaining why studies comparing treatments and outcomes have found better survival rates in migrant groups living in the United States (US) rather than in their country of origin.^4^, ^5^, ^6^ However, they still tend to do worse than the US average.^4^, ^5^, ^6^, ^7^ Cultural differences between the majority (non‐Latin American) white and black and minority ethnic (BME) groups^8^ combine with within‐country ethnic differences in service access^9^ and treatment^10^ to compromise BME group health outcomes and engagement with and trust in health care.^8^ Similarly, in the United Kingdom (UK), white British and BME groups have different diagnosis and postdiagnosis care pathways.^10^, ^11^, ^12^, ^13^ National UK survey data show BME men with PCa express considerable dissatisfaction with care.^14^, ^15^, ^16^ Better understanding of their experiences is needed to improve services, enhance satisfaction, and reduce inequalities^14^ in line with UK government and charity recommendations.^17^, ^18^, ^19^ We have completed a comprehensive metasynthesis of qualitative studies on the postdiagnosis PCa experience for men and their partners.^20^ We made an *a priori* decision to undertake and contrast a subsynthesis in which we consider data on BME men with PCa. To our knowledge, this is the first systematic qualitative review of BME PCa experiences, although there are 2 expert overviews. One considered the qualitative and quantitative literature on African American men's health beliefs regarding PCa, focusing on preventive health‐related behaviors.^21^ The other concentrated on UK BME information and psychosocial support needs.^22^

## 2 Methods

Our metasyntheses are part of a PCa UK/Movember‐funded study.^23^ We use the term “main synthesis” for all studies excluding those considered in the BME subsynthesis, to enable variance between them to be shown. Full methods for our main metasynthesis are described elsewhere.^24^ Here, we report on methods relevant to the subsynthesis. Inclusion and exclusion criteria are shown in Box 1.

**Box 1: Inclusion and exclusion criteria**

*Primary inclusion criterion*:

- Qualitative studies in which at least 50% of analytical themes consider, and include an example of, the PCa illness or management experience for adult men (16 years and older) with PCa and/or their partners or caregivers

*Secondary inclusion criteria:*

- English‐language studies post‐1999
- Empirical qualitative studies (standalone, secondary, or discrete components of mixed method studies) using qualitative methods for both data collection and analysis
- With original data extracts relevant to the synthesis
- Peer‐reviewed published articles or reports

*Exclusion criteria:*

- Men not diagnosed with PCa prior to participation in the research
- Data on diagnosis experiences (explored in detail by others^25^)
- Book chapters, dissertations, and grey literature

*Additional inclusion criterion for the ethnicity subsynthesis:*

- Studies with at least 10% of participants from BME groups

*Additional exclusion criterion for ethnicity subsynthesis:*

- Studies that did not consider or note ethnicity in their findings or attribute extracts by ethnicity

Seven electronic medical, sociological, and psychological databases were searched: MEDLINE, CINAHL, PsycINFO, Web of Science, SSCI, AHCI, and ProQuest IBSS, with backward and forward citation tracking of all included papers. The final search was on 15 December, 2015. Post‐1999 articles only were included, given the widespread adoption of prostate‐specific antigen testing and new treatment regimens and management approaches for PCa since then.^26^, ^27^, ^28^ We included English‐language articles only, because of the issues of translating “meaning” across languages. Our search strategy (Supporting Information) combined terms for PCa with an adapted version of a published qualitative studies search strategy^29^ for optimal sensitivity and specificity.

The lead reviewer (C.R.) undertook initial screening of 1934 identified titles, leaving 711 papers requiring independent assessment, with L.M., of abstracts, and full texts where relevant (in 474 cases), to determine eligibility. Disagreements were resolved by discussion and when necessary (3 papers) adjudication by E.W. or R.W. Study data were extracted by C.R., recording publication information, study design, disease or management details and participant variables, with full replication of 2 key types of data as specified by Noblit and Hare^30^:

1. The literal experiences expressed by study participants in original extracts in the papers (first‐order constructs).
2. Paper authors' interpretations and conceptualizations (second‐order constructs).

Study details were summarized on an Excel spreadsheet. C.R. created preliminary first‐ and second‐order construct lists from the raw data on a separate spreadsheet, using words from the articles, facilitating further analysis. Extraction forms were also uploaded into Nvivo v.10^31^ to manage the metasynthesis. Fifty‐five per cent of included studies were double extracted by L.M., J.N., R.W. and E.W.; extraction differences were successfully resolved through discussion.

### 2.1 Quality assessment

L.M., C.R. and J.N. assessed all studies for quality using a scoring system adapted by C.R. from previous published criteria^32^, ^33^ (Supporting Information), which considered the following:

1. credibility,
2. methodological congruence (including dependability and confirmability),
3. analytical precision,
4. transferability, and
5. heuristic relevance or applicability.

Studies were categorized as “good” (score 18‐24), “fair” (score 12‐17), or “poor” (score 1‐11), with all included^34^ since methodologically weak papers can still provide rich conceptual insights in metasyntheses.^35^

### 2.2 Analysis

We used a modified form of Noblit and Hare's metaethnography approach.^29^, ^36^, ^37^ C.R. applied reciprocal translation^29^ within each Excel constructs list using a matrix with construct names as rows and papers as columns. This involved identifying whether constructs corresponded (“reciprocal synthesis”) or contradicted or challenged each other (“refutational synthesis”) or identified different aspects of the topic under study (a “line of argument synthesis”).^29^ For example, if 1 study reported that BME men avoided disclosing their PCa to others, and another that they educated community members about their cancer, these might be considered refutational and translated into a new construct incorporating elements of both. However, if a third study suggested men found it hard to disclose their cancer initially but shifted over time to community awareness activities, all 3 interpretations would be treated as reciprocal and 2 translated into the one encompassing all 3. C.R. also developed subconstructs; for example, “support” might be subdivided into “instrumental,” “social,” and so forth. These helped reconfirm the construct names in our final list and guide our lines of argument explorations. This process resulted in 2 distinct sets of first‐ and second‐order constructs grounded in the literal and conceptual data of the original papers. L.M. repeated the process for 50% of papers, with the 2 sets compared and differences resolved through discussion and re‐reading of the original papers. C.R. evolved preliminary overarching conceptual third‐order constructs from the final lists of first‐ and second‐order constructs. L.M. repeated the process for a data subset, with the constructs from each analyst compared, discussed, and refined. C.R. checked all third‐order constructs back against (*a*) the first‐ and second‐order constructs to ensure accuracy and sufficiency and (*b*) the original articles. Other authors checked subsets. All authors (from varied professional and ethnic backgrounds) discussed the analysis for rigor and credibility of the final synthesis.

### 2.3 Ethnicity subsynthesis

To explore ethnicity, we used a 2‐tier selection process. We identified studies in which BME groups accounted for 10% or more of participants, and then we excluded those that did not consider or note ethnicity in their findings or attribute extracts by ethnicity. We calculated the manifest effect size for key themes (ie, the proportion of papers, and separately, studies in which the theme was reported).^38^

## 3 Results

There were 184 papers in the main metasynthesis, mostly from Northern and Western Europe, North America, and Australasia. Forty‐two papers were selected in the initial ethnicity filtering; 21 were excluded because of insufficient data (Supporting Information). The remaining 21 papers (13 studies covering 11 ethnic groups) were analyzed.^9^, ^39^, ^40^, ^41^, ^42^, ^43^, ^44^, ^45^, ^46^, ^47^, ^48^, ^49^, ^50^, ^51^, ^52^, ^53^, ^54^, ^55^, ^56^, ^57^, ^58^ The selection flow and study numbers are shown in Supporting Information, and details of the final studies chosen in Supporting Information. Two studies^47^, ^48^, ^52^ considered the Pacific Islands; we included these since according to US census data (http://www.census.gov/topics/population/data.html), less than 14% of Hawaii's population comprises native Hawaiian or Hawaiian Pacific Islanders, and the US white population comprises only 24%. Three studies focused on partners;^9^, ^47^, ^48^, ^54^ in one,^54^ both spouses and men with PCa were interviewed. Studies were identified as good (n = 6) or fair (n = 7) quality.

### 3.1 Constructs

The first‐, second‐ and third‐order constructs explored in this paper are listed in Supporting Information; we consider the third‐order constructs in more detail below. We aimed to evaluate patterns of variance between dominant and nondominant groups and thus do not explore constructs that are largely identical in the ethnicity and main syntheses. These are summarized briefly in Supporting Information and considered in more detail elsewhere.^21^

Among the third‐order construct sets unique to the BME studies was the use of complementary and alternative medicine, discussed in 4 studies,^45^, ^48^, ^53^, ^56^ with a low manifest effect size of 31% of studies, 19% of papers. In 1, a Hawaiian study,^56^ there was no real difference between resident Southeast Asian BME groups and minority “Caucasians,” and authors of another^45^ suggested their findings were similar to those from the wider non‐PCa literature. So we do not consider this construct further but focus only on third‐order construct sets that the primary authors marked out as culturally significant for the BME groups considered.

#### 3.1.1 Spiritual alliances and the development of resilience and empowerment

The aim of 3 BME articles^42^, ^48^, ^50^ was to explore spirituality, or connection to a higher being; in others,^9^, ^39^, ^43^, ^44^, ^45^, ^46^, ^47^, ^49^, ^51^, ^53^, ^54^, ^55^, ^58^ it was an emergent theme. The US and UK African Caribbean and Latin American men frequently spoke of the spiritual, generally in Christian terms.^9^, ^39^, ^42^, ^43^, ^44^, ^45^, ^46^, ^49^, ^50^, ^51^, ^53^, ^54^, ^55^, ^58^ Authors of 1 paper^42^ considered spirituality to be particularly associated with African Americans, tracing this connection historically to the US slave trade. Some Asian women in Hawaii^48^ observed ethno‐cultural spiritual traditions such as ancestor worship in parallel, or were Buddhist or Taoist, or used meditation as a spiritual force. Study participants' beliefs were often strengthened by their cancer experience,^42^, ^46^, ^50^, ^51^, ^54^, ^55^ with men actively seeking out the spiritual to help them cope,^42^, ^51^ and an increase in church attendance reported for affected couples.^54^, ^55^ The impact of God on men's views of their own agency in coping emotionally with their experiences and dealing with their cancer and its consequences varied.

Some men left everything to fate or God's will,^42^, ^49^, ^50^, ^51^, ^54^, ^58^ which was also reported by some partners.^9^, ^47^, ^48^ This was more likely to be explicitly stated by Latin Americans than by African Americans.^49^ Whether the outcome was positive or not, they felt resigned to or even comfortable with this. In these accounts, the men developed resilience but were not agentic in management of their health. Responsibility was transferred to a higher power:

I know that it's not gonna spread any faster than God will let it spread. … If God's will is to make me better or get rid of this cancer, or it may just linger with me, it doesn't matter to me because I know it's His will.^54^

Such an approach could be linked to fatalism^47^, ^49^, ^50^ or a father‐child relationship^42^ but was sometimes considered by authors as a collaboration or partnership formed between men or their partners and God “to hold together a self‐image to live with.”^39^, ^48^ Spirituality in these accounts transformed men's views of their bodies from wounded by cancer to hosting cancer.^39^, ^50^ Overall, this enabled men to focus on living rather than dying but could lead to a seeming rejection of health care; as 1 man said: “if you got Jesus on your side, the health and life insurance, you don't need it.”^54^ Similarly a partner said: “I put him in the hands of God so that He could heal him.”^9^

Given that many US BME men were ineligible for health insurance,^50^ this might simply indicate a mechanism for developing resilience in coping with their economic circumstances and limited access to care. This is indicated by the more pragmatic approach of some men: “I think prayer helps you relax and gives you peace of mind.… I don't think it prevents or heals cancer.”^45^

Authors of 1 study^42^ suggested African American men were unique in their descriptions of a *personal* relationship with God, who appeared to them during moments of crisis, or otherwise provided individualized support: “What took my fear away was the fact that I believed that God would not put any more on you than you can handle.”^42^ In such cases, spirituality could be seen as complementing health care. Study authors said these men did not need support from church or community activity, a throwback to the restrictions of slavery;^42^ however, other studies highlighted the importance to men of their church community.^50^, ^54^, ^55^

Other men in the studies (and all the men in 1 study^45^) described a triumvirate of God, the clinician, and patient, with God giving the other 2 the means and skills to do their part as God's instruments.^42^, ^43^, ^44^, ^45^, ^46^, ^49^, ^50^, ^51^ All 3 were agentic within this alliance: “So I can't say God did it all by himself or whatever. I think, to me, he give doctors knowledge”^45^ and “you got to help God, you just can't depend on God to do everything.”^45^ As Maliski et al^50^ commented, “God had his role, the physician had his/her role, and the patient had a role to play in a successful treatment and recovery.” The men in this group felt empowered to actively overcome the challenges of cancer, for example, by actively participating in and cooperating with treatment, rather than surrendering passively to fate.^50^

Although most BME men—and if studied, their partners—embraced spirituality, some did not, relying rather on social support.^39^, ^46^ Authors of 1 study suggested spirituality was stronger in men embedded in their traditional communities in the US “Bible belt,”^46^ highlighting its cultural significance, although they were uncertain about transferability of their findings.

Spirituality was reported in the main metasynthesis in 4 studies.^59^, ^60^, ^61^, ^62^ All 4 reported that Caucasian men sometimes drew comfort from spirituality; 2 studies 60, 61 said some prayed for cure and derived companionship support from church attendance (although some eschewed religious institutions) or even just from talking to God. However, none mentioned a spiritual alliance (partnership, collaboration, or triumvirate) with God.

#### 3.1.2 One more thing in the lifelong fight against adversity

Men and their partners from a range of BME groups^9^, ^47^, ^48^, ^58^ described significant adversities through their lives, linked directly or indirectly to their BME status. Life was portrayed as a perpetual war, lived in impoverished circumstances of inequality. Thus, partners in Hawaii spoke of absent fathers working abroad, or families on the US plantations, or internments, if Japanese, after Pearl Harbour.^47^ African American men spoke of a threat‐filled ghetto lifestyle.^58^ Authors of 1 study^47^ reported that almost 75% of participants described “ethnic‐specific” adversities. Both participants and authors of the different studies reported these to be transformative and empowering; the authors found they imbued men and their partners with learned skills, resilience, and the strength to fight and normalize the cancer as they had done with other adversities.^9^, ^47^, ^48^, ^52^, ^58^ This attitude was traced back by African American men^50^, ^58^ and Japanese and Chinese partners^47^, ^48^ to cultural models as well as lived experience. Cancer, like life, was referred to using battle metaphors; although this is common in the cancer literature,^63^ its connection with cultural inequalities is not. While phrases such as “fighting spirit”^58^ and “standing up to cancer”^58^ were used alongside occasional admissions of a failure to do this and of feeling “defeated”^9^ by the cancer, Zhang et al^58^ used quantitative methods to show that reports of adversity were significantly correlated with a lack of fear of cancer.

Within the main metasynthesis, men from the dominant white groups often coped by normalizing their cancer as just another life event. But they referred only to the every day, or to other illnesses, and not to adversity as described above or to cultural role models of resilience.

#### 3.1.3 Cultural differences in male self‐identity and the phenomenon of shifting masculinities

Erectile dysfunction (ED) caused by the cancer or its treatment^64^, ^65^ was a particular challenge to men's masculine self‐image.^39^, ^40^, ^41^, ^49^, ^54^, ^55^ An apparent magnified significance was attached to sexual dysfunction in some ethnic groups compared with Caucasian men, although these also experience considerable psychosexual distress.^67^, ^68^ The magnified impact was mentioned in 2 studies only in the main metasynthesis, set in Turkey^69^ and Israel.^70^, ^71^, ^72^ Significantly, evidence of survival was considered only in the same ethnic groups (BME,^50^, ^51^, ^54^ Turkish,^69^ and Israeli^73^) to be marked by the cessation of management of treatment side effects, including use of Viagra,^50^, ^51^, ^54^ which may be related to such attitudes.

Explicit examples of the magnified sexual impact include a participant declaring “nothing so important apart from that to an African man”^41^ and authors referring to a “culture fraught with sexual competition and oneupmanship.”^40^ Matheson et al. (Matheson, Watson, Nayoan, Wagland, Glaser, Gavin, Wright & Rivas, submitted) found a similar, although not identical, pattern in another of our subsyntheses, in young, unpartnered and gay men. This accords with Connell's^66^ theory of subordinated masculinities. The difference was confirmed in a study comparing Caucasians and African American men,^40^ one from England comparing 'British', Irish, Asian and African men,^41^ and a study comparing Latin Americans and African Americans.^49^ Authors of this last study commented that the different ethnic groups had similar values but enacted them differently and to different intensities “within their own sociocultural contexts, and were influenced by early cultural influences.”^49^ Illustrative of this, Gannon et al^41^ reported African American men as believing there was “nothing so important” as being sexually active and an Asian man as declaring its unimportance as he was “not a teenage boy.”

Initially, men adjusted in different ways to the problem. The US Latin American men prioritized partner bonding over the need to prove masculinity through sexual conquest.^49^ This was also broadly typical of men in the main metasynthesis, but Latin Americans also focused on their role as family provider for validation of their masculinity; if this role was maintained and they had children, the ED was less problematic.^49^ A similar focus was only reported in 1 paper from Brazil^74^ and 1 from Israel^70^ in the main metasynthesis. African Americans rooted manhood irrevocably in sexual prowess,^40^, ^49^, ^54^ unlike men in the main metasynthesis. They used sexual aids to cope (which the Latin American men avoided) and reported this notion of manhood only restricted *other* men from their community, who had avoided treatment for *their* PCa^40^ as “they felt they did not have a useful life left once that [sex] was taken away.”^40^

To cope in the longer term with ED, all men eventually reframed the concept of manhood. They shifted priorities away from sexuality^39^, ^41^, ^47^, ^48^, ^49^, ^54^, ^55^ to the relational,^49^ so they felt different to other men, but ultimately no less of a man^39^, ^40^, ^47^, ^49^, ^54^ (1 study excepted^41^). Many BME men and their partners managed this by normalizing the situation as part of ageing,^9^, ^39^, ^41^, ^47^, ^49^, ^51^, ^52^, ^53^, ^54^ which was also promoted by doctors.^53^ This process of shifting multidimensional and socially negotiated masculinities,^49^ also common within the main metasynthesis, is well established in the broader sociological literature.^66^

#### 3.1.4 Cultural pressures to maintain a social front that conceals

The BME men talked about maintaining a strong “front” to others^40^, ^49^ early in their cancer journey, tending to only disclose the cancer selectively within their immediate networks.^47^, ^49^, ^50^, ^54^, ^58^ While common also in the main metasynthesis, this was considered by primary study authors to be a particular issue in BME groups,^47^, ^49^, ^54^, ^58^ something noted in general for African and Caribbean people,^75^ and with divergent reasons between the BME subgroups.

For the Latin Americans, selective disclosure was intended to protect their family from fielding difficult interactions (eg, pity and stigmatization).^49^ The African Americans and Afro‐Canadians valued their sexual “bragging rights” as part of their identity,^40^, ^49^ so talked with friends as if still sexually active.^49^ The stigmatization of cancer, magnified masculinity issues, and a community‐facing culture^76^ conspired to silence the men. Thus, it was reported: “cancer is particularly stigmatised and the fear of social rejection is particularly high in African‐Americans compared with white Americans”^58^ (see also literature^40^^,^ 49). A silencing stigma was also described in 7 studies in the main metasynthesis,^69^, ^70^, ^71^, ^72^, ^74^, ^77^, ^78^, ^79^, ^80^ but in 3, at least 10% of participants were black,^77^, ^78^, ^79^ (excluded from the synthesis as they did not report data by ethnicity, see Supplementary information) and the others were set in Brazil,^74^ Turkey,^69^ and Israel.^70^, ^71^, ^72^

Nondisclosure strategies could be harmful rather than protective, by increasing participants' emotional burden and blocking support.^47^, ^54^ In 2 studies,^47^, ^55^ partners encouraged men to talk more to others by explaining that their secrecy reduced access to the support they needed;^55^ making jokes;^47^, ^55^ normalizing;^47^, ^55^ and giving men moral support by accompanying them to support groups, with gaze suitably lowered during sensitive talk.^47^ Ka'opua et al stated that these approaches might be considered “indirect by Western standards,”^47^ linking them firmly with community‐facing cultures.

Participants across the main and ethnicity syntheses reported the value to well‐being of social support from close networks,^9^, ^39^, ^42^, ^43^, ^44^, ^46^, ^47^, ^48^, ^49^, ^50^, ^52^, ^53^, ^54^, ^55^, ^58^ once they had moved past barriers to disclosure (see Supporting Information).

#### 3.1.5 Surviving for others and a legacy after death

With time, men re‐evaluated their life priorities, developing the desire to warn others about the disease.^39^, ^42^, ^50^, ^52^, ^53^, ^54^, ^55^ Their primary focus, in “giving back,”^47^, ^50^ was to redress the lack of awareness among men from their ethnic community.^9^, ^46^, ^52^, ^53^, ^55^ Similar behaviours were found in the main synthesis, and cut across the groups in a mixed ethnicity study including white partners/couples.^52^However, men in the main metasynthesis were more likely to be inward looking when reframing life priorities, increasing the time spent on the things they enjoyed in life rather than helping others.

The men from BME groups drew overtly on their faith or spirituality and a community‐facing attitude when describing their activities. There were 3 ways this was done, specific to the BME metasynthesis.

First, some men considered God had given them cancer as a test, or allowed them to survive, precisely so they would set new priorities in life to fulfill their purpose:^9^, ^39^, ^42^, ^44^, ^47^, ^48^, ^49^, ^50^, ^51^, ^52^, ^53^, ^54^ “I thought God has me here for a reason, so I'm back with God and talking to men about getting PSA tests.”^50^ They linked their spiritual growth with their community‐facing cultural attitudes to sharing and caring,^47^ “doing unto others,”^44^ and exchanging or brokering information,^44^, ^51^, ^52^ activities that were specifically linked by study authors to their BME status. This refocus of attention onto others was suggested to function as a form of positive denial.^39^ Whether or not this was so it helped men cope, providing them with emotional capital^44^ as social capital is known to do.^81^ Partners were sometimes involved,^47^, ^48^ benefitting from affirmation of their spiritual connection, cultivation of a sense of purpose, and integration of their own experience of cancer.^48^

Second, men often spoke of the afterlife, highlighting their fear of cancer recurrence and concerns about their own mortality,^42^, ^50^, ^51^, ^54^ which their partners shared.^9^ The afterlife was not mentioned in the main metasynthesis, although fears of recurrence and death were. Some BME men hoped to go to a “paradise,” although others were uncertain as to what, if anything, lay beyond death.^50^ Authors suggested that beginning—or in some cases increasing— spiritual, PCa awareness and charitable activities enabled men to leave a legacy and hence achieve some sort of immortality or believe they had secured a better place in the afterlife.^9^, ^39^, ^42^, ^43^, ^44^, ^47^, ^48^, ^49^, ^50^, ^51^, ^53^, ^54^

Third, men sometimes bargained with God, vowing to “becom[e] more involved in the church,” “set a better example for men in the neighbourhood,” or “educate other men about PCa” in return for survival.^50^ This could give a sense of invincibility: “God has kept me here for something and until I have done what He wants me to do, He's not going to take me.”^42^ These nuances were not mentioned in the main metasynthesis, although there is no reason to believe non‐BME men do not sometimes think this way.

The men made positive changes in health behaviors,^9^, ^51^, ^54^ which some authors suggested was simply to live long enough to set their affairs in order. Then what they had begun with their families—and therefore their presence—would continue were they to die.^47^, ^50^, ^51^, ^53^, ^54^ But it might also be so they could live for their family.^47^, ^50^, ^51^, ^53^, ^54^ Not all dietary changes were family focused, with some a simple response to symptoms and treatment side effects: “You have to watch what you eat now because of your bowels and stuff, they …sometimes don't act right.”^55^ Exercise was sometimes reduced rather than increased, when men felt drained and weak.^55^ Partners often drove the changes^9^, ^48^ for the men's sake, initially often meeting resistance.^9^

Improved health behaviors and caretaking of these by partners were also found in the main synthesis, but family‐focused rationales were not. Moreover, Asian participants in 1 study enjoyed spiritual beliefs and practices such as Tao‐chiao that were directly associated with physical health,^48^ not reported in the main synthesis. Authors of 1 study^55^ noted healthy behaviors were harder for men from BME groups to achieve because of the lack of culturally appropriate information, something noted for other long‐term conditions such as diabetes.^82^, ^83^

#### 3.1.6 The relationship with health care providers

There was little difference between men in the BME and main metasyntheses concerning their relationships with and views of their health care providers, but our subsynthesis revealed important nuances. Significantly, although BME groups are often said to prefer a relatively patriarchal form of medicine over shared care,^57^ overall, the men showed little evidence of this.^45^, ^57^ Indeed, many checked out their doctor's competence before surgery, seeking those with a reputation for preserving erectile function.^39^, ^40^ This was absent from the main metasynthesis and links to the magnified impact of ED in some BME men. Many BME men had a particular need for dialogue with their health care professional (HCP) because the stigma of having cancer and ED blocked their help‐seeking within their community networks.^57^ The same stigma could ironically obstruct patient‐centered care, making conversations with their HCP difficult.^49^ Participants considered delicate conversations would benefit from a communication triangle involving the man, his partner, and HCPs together.^54^ Participants reported a lack of respect and empathy from clinicians.^40^, ^49^, ^53^, ^57^, ^58^ This was also found in the main metasynthesis, but authors of 2 studies^53^, ^57^ commented on its criticality in exacerbating BME groups' general mistrust of health care. Cultural communication subtleties were also described, for example, participants could feel insulted if addressed by their forenames in a clinical setting.^57^

#### 3.1.7 A lack of economic capital

The financial impact of PCa was evident across the BME and main syntheses inasmuch as it affected men's employment.^53^, ^58^ Only US BME papers^9^, ^46^, ^50^, ^52^ included talk about financial and physical stresses of treatment costs and access to services caused by a more endemic economic disadvantage. Thus, authors of 1 paper^50^ stated “as uninsured minority men, they did not have the options and resources available” to white middle‐class Americans. In 1 study,^52^ minority Caucasians in their Hawaiian sample were similarly affected. Several participants in another study^46^ commented on the value of health care insurance in decreasing the financial strain, which they perceived as a particular issue for African Americans. Williams et al^9^ noted other structural barriers but provided no illustrative extracts, commenting that the need for emotional support was over‐riding, as evidenced in other papers from their study^49^, ^50^ and a different study.^58^ Similarly, in a UK study,^53^ the intersection of social class (as a proxy for financial capital), age, and ethnicity were explored; ethnicity remained the strongest factor.

## 4 Discussion

This is the first study to systematically draw together the qualitative literature on the BME PCa experience. Our search was comprehensive yet found little consideration of ethnic similarities and differences between white and BME groups, even when studies sampled across ethnicities. Most BME studies were undertaken in the United States, with results that may not be transferable to other countries with other histories and health systems, as suggested, for example, by findings reported for the “lack of economic capital” construct.

We found that BME men's reports mostly differed from those of the dominant white groups in the main metasynthesis in their intensity or in the nuanced detail, and that the authors of the BME studies sometimes overemphasized the differences they found. We also found partner experiences accorded with those of the men. The intersection of migration, social class, education, historical, and cultural factors with men's experiences was influential in shaping the men's experiences and behaviors.^53^

**Figure 1:**
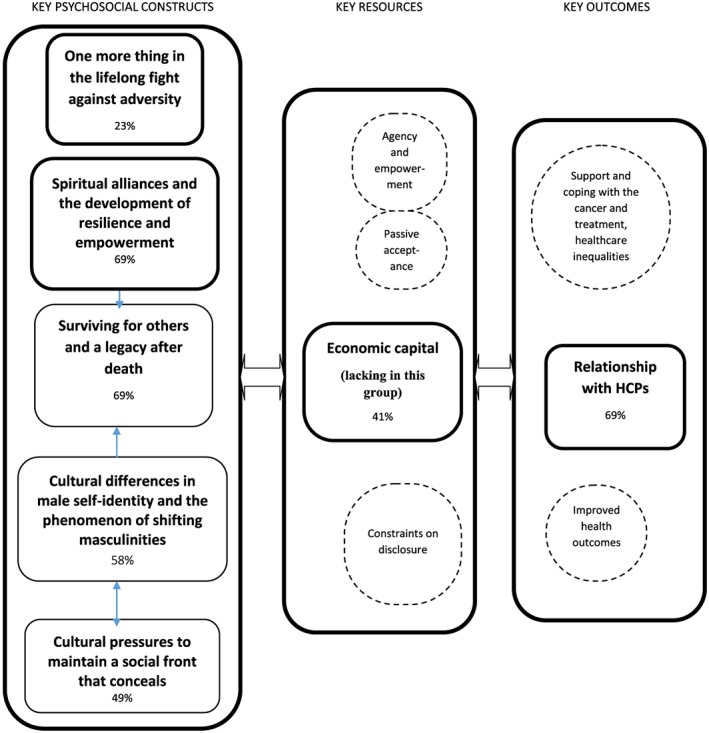
Logic model to show how the third‐order constructs interconnected. The constructs are in solid line boxes, and consequences of these in circles with dotted lines

An important construct (study effect size 58%) (Figure 1) concerned the magnified impact ED had for men from some BME groups. Their threatened masculinities intersected with the stigmatization of cancer and a fear of social rejection, to compromise perceived social identities, particularly marked in African Americans. Initially, men put on a macho “front” as a form of impression management^84^ and avoided talking about cancer and their ED with people outside their immediate families. In so doing, they blocked support from their community; such nondisclosure is known to increase patients' and carers' psychosocial burden.^9^, ^85^, ^86^ There is a recognized need for better psychosocial support for men with PCa from BME groups^22^ and more generally.^87^ Participants' initial secrecy made them particularly reliant on a good relationship with HCPs. However, the men were often dissatisfied with this (study effect size 69%), which is recognized in the broader literature as a particular problem for BME groups.^14^, ^15^, ^16^, ^17^, ^18^, ^19^, ^88^ In general, HCPs need to develop more culturally competent communication skills.^46^, ^53^, ^88^ Encouragingly, many men slowly developed an interest in engaging in their own care,^57^ which is typical of patients with long‐term conditions including cancer who gradually become experts in their condition.^89^ This suggests enhanced HCP communication would be particularly productive in the post‐treatment phase.

The BME men and their partners described ethno‐cultural experiences of adversity (effect size 23%) as imbuing them with resilience and emphasized the importance of spirituality to coping. In particular, spiritual beliefs and alliances with God (study effect size 69%) empowered men through their cancer journey and enabled them to transcend health care issues, inequalities, and a lack of economic capital.

Over the longer term, men were able to adjust to cancer‐ and treatment‐related issues and reframe their masculinities in ways that separated the relational from the physical or that normalized their problems as due to age. This was identical in the main metasynthesis. This may suggest that once men can break through cultural barriers, and as they move through health care, their coping mechanisms begin to approximate those of the dominant groups. Empowerment intersected with thoughts of mortality and life's purpose, spiritual growth, and a community‐facing cultural attitude, leading them to do good works within their communities (study effect size 69%), whereas men in the main metasynthesis responded to similar thoughts by focusing on life's pleasures.

The men's community advocacy helped them draw on community networks of support; a reciprocating relationship was developed^40^, ^44^ that services could consider when modeling interventions.^8^ Although an association between spirituality and financial capital was not shown in a large US cross‐sectional survey,^90^ reciprocating community relationships and increased spirituality as coping mechanisms may be associated with and compensate for reduced financial capital and hence reduced access to health care support.^40^, ^44^, ^50^ An intervention tapping into both might be significant in reducing inequalities. As a start, the English Department of Health has explicitly referred to “voluntary sector ‘buddying’ schemes and community outreach [which] were regarded as particularly important to connect with BME communities and for those communities to connect to services.”^91^, ^92^ Despite this, much remains to be done; we know of only 1 published intervention designed to support men with PCa from BME groups specifically,^93^ with another US e‐technology intervention study in process (http://grantome.com/grant/NIH/R01‐MD007783‐01A1).

Our findings show the importance of recognizing that all social identity and self‐identity work is complex, intersectional, and constantly in negotiation^94^ and that static cultural stereotyping is not helpful.^53^ They also suggest that if health care services are able to support men from BME groups in their reframing of their identities, drawing where appropriate on spiritual beliefs, there will be community as well as individual benefits. More studies are needed in diverse ethnic groups^95^ to confirm and build on our findings and inform the design of further interventions. Studies should develop aims that address clinically significant gaps in knowledge.

### 4.1 Limitations and strengths

Our study has several limitations. “Ethnicity” is a problematic concept,^95^ with considerable heterogeneity between individuals in how they perceive their own and others' ethnicity, heterogeneity within overarching ethnic labels such as African American, and intersection with other factors such as socioeconomic status. However, our focus proved useful in revealing important patterns. The studies themselves were conceptually and methodologically heterogeneous. We tried to identify and synthesize all relevant qualitative literature and therefore included an analysis of survey freetext^52^ and a focus group‐based study^46^; however a sensitivity analysis showed their removal would not affect overall findings. The locations of and varying aims of the studies, as restricted by what was available, undoubtedly led to bias in our reporting and may have led to the risk of stereotyping.^96^ One study^39^ reporting spirituality recruited its participants through a church social worker, while 3 studies^39^, ^42^, ^53^ included church ministers in their samples. This suggests a bias that often occurs when studies of BME groups recruit from churches or close‐knit community groups. However, the other studies detailing recruitment used patient lists and so were not inherently biased to the spiritual. This suggests our findings are robust and demonstrates an advantage of metasynthesis in collecting studies together. There is potential for some publication bias, although we tried to correct for this by including manifest effect sizes based on study numbers. Different quality criteria might have scored studies differently relative to each other, and older studies are often disadvantaged in quality criteria scoring owing to temporal differences in study design and reporting requirements.

Our study has many strengths. The metasynthesis was rigorous, involving clear criteria, an experienced team, and various quality checks, with indications of effect sizes and quality. Our approach was systematic, and our inclusion criteria were specific. We aimed to ground our analysis in the papers' findings, although it remains possible our own perspectives and backgrounds influenced interpretations. Comparing findings with our main metasynthesis enhanced study dependability.

## 5 Conclusions

The PCa experience of men and their partners from BME groups is similar to that of dominant white groups. But culture, ethnicity, history, and demographics often contribute to a complex of intersecting factors that create nuances in the BME groups' experiences and behaviors. Health care for PCa should consider and harness men's contextually and culturally specific coping mechanisms, for community as well as individual benefits. Services should avoid cultural stereotyping and, while acknowledging difference, be open to the negotiation of changes in felt masculinities, social identities, and spiritual beliefs. More studies are needed in diverse ethnic groups, and with aims that target significant gaps in knowledge, to reduce inequalities.

## Conflicts of interest

None.

## Sponsor

This study was funded by Prostate Cancer UK in partnership with Movember (grant number: HO‐LAPCD‐14‐001), and sponsored by the University of Leeds.

## Supporting information

Supporting info item

Supporting info item

Supporting info item

Supporting info item

Supporting info item

Supporting info item

Supporting info item
