# Supplementary material for: Ethnicity and the prostate cancer experience: a qualitative metasynthesis
Source: Psychooncology. 2016 Aug 23;25(10):1147–56. doi: 10.1002/pon.4222 (PMC5096040; doi:10.1002/pon.4222)
Supplement: Supplementary file 1 — Supporting info item [file PON-25-1147-s001.docx]

**Suppl file 1: Basic search, amended for different databases**

Medline (Ovid) search strategy:

((qualitative or qualitative adj (study or studies or research)) or (ethnograph* or

phenomenol* or observation* or naturalistic or lived adj experience* or life adj

experience* or perspective* or attitude* or belief* or theme* or thematic) or (content

adj analys* or discourse adj analys* or thematic adj analys* or narrative adj analys*) or

(field adj (study or studies or research)) or (purposive adj sampl* or purposeful adj

sampl* or theoretical adj sampl*) or (grounded adj (theor* or study or studies or

research)) or (constant adj (comparative or comparison)) or (focus adj group* or

interview* or participant adj observ*) or (emic or etic or hermeneutic* or heuristic or

semiotic*) or (data adj saturat*) or (text adj mining) or (keyword adj analysis)).tw *AND*

(((prostate or (prostat$ adj5 cancer$) or (prostat$ adj5 neoplas$) or (prostat$ adj5

carcinoma$) or (prostat$ adj5 tumo?r$)).tw) OR exp prostatic neoplasms/)

Other databases, Web of Science, Cinahl and Psycinfo searched using simpler strategies

based on: TX qualitative research AND (TX prostate cancer OR SU prostate cancer).
