# Supplementary material for: Ethnicity and the prostate cancer experience: a qualitative metasynthesis
Source: Psychooncology. 2016 Aug 23;25(10):1147–56. doi: 10.1002/pon.4222 (PMC5096040; doi:10.1002/pon.4222)
Supplement: Supplementary file 2 — Supporting info item [file PON-25-1147-s002.docx]

Suppl file 2: Quality assessment of included papers for LAPCD meta-synthesis

Read the qualitative study/extraction sheet and score each of the categories listed using the quality rating scale of 0/1 through 3 described below (adapted from Cochrane recommendations and Cesario et al, 2002).

| Study details |  | | |
| --- | --- | --- | --- |
| 1. Descriptive vividness for truth value: Credibility | |  | |
| e.g. validation by others (data groups etc), member validation, peer debriefing, attention to negative/’deviant’ cases, independent analysis of data, verbatim quotes, persistent observation (longitudinal), saturation of themes/theory | | Each one scores 0.5 (max score 4) | Tally |
|  | | 3 = Good (tally of 3-4)  2 = Fair (tally of 1.5-2.5)  1 = Poor (tally of 0.5-1)  0 = (tally of 0) | Score |
| 2. Methodological Congruence | |  |  |
| 2a: method description | |  | |
| 1. Study questions or aims are identified | | scores 0.5 | Tally |
| 1. Philosophical/theoretical framework is made explicit | | scores 0.5 |  |
| 1. Limitations are identified (properly, not token) | | scores 0.5 |  |
| 1. Site described fully | | scores 0.5 |  |
| 1. Sampling approach described fully | | scores 0.5 |  |
| 1. Population described fully | | scores 0.5 |  |
| 1. Data collection approach described fully | | scores 0.5 |  |
| 1. Data analysis method described fully | | scores 0.5 |  |
|  | | 3 = Good (tally of 3- 4)  2 = Fair (tally of 1.5-2.5)  1 = Poor (tally of 0.5-1)  0 = (tally of 0) | Score |
| 2b. Procedural rigor and consistency: Dependability: | |  | |
| e.g. peer review, audit trails, triangulation in the context of the use of different methodological approaches, reflexivity of the research process and use of theory/literature, calculation of inter-rater agreements etc. | | Each one scores 1 (max score 4) | Tally |
|  | | 3 = Good (tally of 3- 4)  2 = Fair (tally of 2)  1 = Poor (tally of 1)  0 = (tally of 0) | Score |
| 2c ethical rigor | |  | |
| Was there mention of ethics approval | | no=0,yes =1 | Tally |
| Was there further consideration of ethical dilemmas | | no=0,yes =1 |  |
|  | | 3 = Good (tally of 2)  2 = Fair (tally of 1)  1 = Poor (tally of 0)  (since current research requires ethical review it has been decided that a zero score (not zero tally) can be discarded as historical and newer studies cannot be compared in the same way) | Score |
| 2d Confirmability (auditability, neutrality) So that other researchers could arrive at similar conclusions if they applied the same biases | |  | |
| e.g. reflection on biases, providing relevant background information on the researcher, were the records of the raw data sufficient to allow judgments to be made? | | Each of the three main points to score 1 | Tally |
|  | | 3 = Good (tally of 3)  2 = Fair (tally of 2)  1 = Poor (tally of 1)  0 = (tally of 0) | Score |
| 3: Analytical Preciseness: conceptualisations developed appropriately? | |  | |
| Did the interpretive theoretical statements correspond with the findings or is there a leap of faith? | | score 0 or 1 | Tally |
| Is there a clear process from descriptive to more conceptual themes? | | score 0 or 1 |  |
| Was a theory or model developed that correctly represents the themes? | | score 0 or 1 |  |
| Is the numerical value of terms such as ‘most’, ‘some’, ‘sometimes’ etc clear? | | score 0 or 1 |  |
|  | | 3 = Good (tally of 3- 4)  2 = Fair (tally of 2)  1 = Poor (tally of 1)  0 = (tally of 0) | Score |
| 4: Theoretical Connectedness to enable application: transferability | |  | |
| e.g. sufficient details of the study participants to enable readers to evaluate relevance to other groups, sufficient contextual background information (setting etc); detailed description of research approach* | | Each of the three main points to score 1 | Tally |
|  | | 3 = Good (tally of 3)  2 = Fair (tally of 2)  1 = Poor (tally of 1)  0 = (tally of 0) | Score 2 |
| 5. Heuristic Relevance | |  |  |
| Are there policy and research recommendations? | | score 1 for each and an extra point if policy recommendations are longer than one short paragraph | Tally |
|  | | 3 = Good (tally of 3)  2 = Fair (tally of 2)  1 = Poor (tally of 1)  0 = (tally of 0) | Score |
|  | | **TOTAL SCORE** |  |

*In methodological rigor the criterion simply considers whether this is stated or not

# FINAL QUALITY OF EVIDENCE RATING (score range 1-24)

The final quality of evidence rating based on the total scores for each of the five categories described above should be calculated as follows for each qualitative study:

1. Good: Total score of 18-24 indicates that 75% to 100% of the total criteria were met.
2. Fair: Total score of 12–17 indicates that 50% to 74% of the total criteria were met.
3. Poor: Total score of less than 12 indicates that less than 50% of the total criteria were met.
