# Supplementary material for: Ethnicity and the prostate cancer experience: a qualitative metasynthesis
Source: Psychooncology. 2016 Aug 23;25(10):1147–56. doi: 10.1002/pon.4222 (PMC5096040; doi:10.1002/pon.4222)
Supplement: Supplementary file 3 — Supporting info item [file PON-25-1147-s003.docx]

**Suppl file 3. Summary of excluded studies.**

| **Study** | **Men with Pca (n)** | **Partners (n)** | **Time since diagnosis in months (range)** | **Age in years; m=mean, md=median, SD= standard deviation, (range)** | **Ethnicity (%)** | **Setting** | **Data collection** | **Data analysis** | **Aims (PCa = prostate cancer)** |
| --- | --- | --- | --- | --- | --- | --- | --- | --- | --- |
| **STUDIES EXCLUDED BECAUSE ETHNIC GROUPS ARE NOT ANALYSED SEPARATELY** | | | | | | | | | |
| Ames, S. C., Tan, W. W., Ames, G. E., Stone, R. L., Rizzo, T. D., Jr., Heckman, M. G., Crook, J. E., Clark, M. M., Rummans, T. A. & Werch, C. E. 2008. Quality of life of men with biochemical recurrence of prostate cancer. J Psychosoc Oncol, 26, 17-34. | 28 | 0 | ns | Md 76 (58-87) | Not Hispanic or Latino 89% Hispanic or Latino 4%  Unknown 7%  Racial origin:  White 86% Black 14% | community via oncology clinic and newspaper adverts, US | Focus groups | Thematic | To determine the unmet psychological needs of men with biochemical recurrence of PCa to guide the development of a QoL intervention |
| Bailey, D. E., Jr., Wallace, M. & Mishel, M. H. 2007. Watching, waiting and uncertainty in prostate cancer. J Clin Nurs, 16, 734-41. | 10 | 0 | <12 (4-12) | (64-88) | White 80% African Caribbean 20% | Urology clinic in tertiary care medical centre, UK | Semi-structured interviews | Thematic; content analysis, then Mishel’s theory as organising framework | To explore the problems and uncertainties of older men with PCa and the strategies they used to manage these problems and uncertainties after undergoing WW |
| Bokhour, B. G., Clark, J. A., Inui, T. S., Silliman, R. A., & Talcott, J. A. 2001. Sexuality after Treatment for Early Prostate Cancer: Exploring the Meanings of “Erectile Dysfunction.” Journal of General Internal Medicine, 16(10), 649–655. | 48 | 0 | ns | (50-79) | White, African-American, proportions ns | Medical centre, staff model health maintenance organization, US | Focus groups | Grounded theory (Glaser & Strauss, 1967) | To explore perceptions of the impact of ED on men who had undergone definitive treatment for early non-metastatic PCa |
| Bowie, J. V., Sydnor, K. D., Granot, M. & Pargament, K. I. 2005. Spirituality and coping among survivors of prostate cancer. Journal of Psychosocial Oncology, 22, 41-56. | 29 | 0 | 1mnth-19 years | From larger study (n=38): M 66 (49-79) | White 59% African Caribbean 41% | Community via support groups, flyers, newspaper advertisements, worth of mouth, radio talk show, UK | Semi-structured interviews with vignettes + focus groups | Thematic; ‘modification of framework approach’ | To elicit beliefs about religion and spirituality among African American and White men with PCa |
| Chambers S.K., Lowe A., Hyde M.K., Zajdlewicz L., Gardiner R.A., Sandoe D. & Dunn J. (2015) Defining young in the context of prostate cancer. Am J Mens Health, 9(2), 103-114. | 15 | 0 | >36 | (41-70) | 80% from Australia, 20% from elsewhere | Patients from major urban centres and regional towns | Semi-structured interviews | Hermeneutic (Interpretive) phenomenology | To draw on the experiences of men with PCa as opposed to an a priori categorization based on chronological age, with chronological, life course, and masculine identity approaches to defining “young” contrasted |
| Eziefula, C. U., Grunfeld, E. A. & Hunter, M. S. 2013. 'You know I've joined your club… I'm the hot flush boy': A qualitative exploration of hot flushes and night sweats in men undergoing androgen deprivation therapy for prostate cancer. Psycho-Oncology, 22, 2823-2830. | 19 | 0 | ns | M 68.6, SD 9.4 (45-84) | White 79%  Black 21% | Recruited from PCa clinics, support groups, a pilot service for PCa survivorship within a hospital trust, UK | Semi-structured interviews | Thematic; framework approach | To explore flush-related experiences in men with PCa as starting point for establishing cognitive appraisals and behavioural reactions |
| Galbraith, M. E., Hays, L. & Tanner, T. 2012. What men say about surviving prostate cancer: Complexities represented in a decade of comments. Clin J Oncol Nurs, 16, 65-72. | 401 | 364 | ns | Men M 68; Partner M 64 (Galbraith ME, Pedro LW, Jaffe AR, Allen TL.  Describing health-related outcomes for couples experiencing prostate cancer: differences and similarities. Oncol Nurs Forum. 2008 Sep;35(5):794-801. doi: 10.1188/08.ONF.794-801) | White 84% | Community survey -Tertiary care non-profit medical centre (Galbraith et al, 2008) | Two open questions from survey | Thematic | To describe the experiences reported by men, in their own words in a 10-year time period, who have been treated for early stage PCa |
| Grunfeld, E. A., Drudge-Coates, L., Rixon, L., Eaton, E. & Cooper, A. F. 2013. "The only way I know how to live is to work": a qualitative study of work following treatment for prostate cancer. Health Psychol, 32, 75-82. | 50 | 0 | <12; M 39 days (SD 20.2) | M 59 (SD 5.81) | White 76% Black 18% Asian/Chinese 6% | Outpatient clinics in hospitals , UK | Semi-structured interviews | Thematic; framework approach | To explore the meaning of work, and linkages between masculinity and work following PCa treatment |
| Grunfeld, E. A., Halliday, A., Martin, P. & Drudge-Coates, L. 2012. Andropause syndrome in men treated for metastatic prostate cancer: a qualitative study of the impact of symptoms. Cancer Nurs, 35, 63-9. | 21 | 0 | ns | M 78 (68-92) | White 66%  Black 24% Asian/Chinese 10% | Hospital clinic, UK | Semi-structured interviews | Thematic; framework approach | To explore the experience and impact of andropause symptoms (particularly hot flashes) among men being treated with ADT for metastatic PCa |
| Jackson, T., Davis, K., Haisfield, L., Dawson, D., Lynch, J., Regan, J., Kwart, A., Lynch, B. & Taylor, K. 2010. Disclosure of diagnosis and treatment among early stage prostate cancer survivors. Patient Education and Counseling, 79, 239-244. | 35 | 0 | >36 | M 64.7 (SD 8.7) | White 43% African-American 57% | Urology department at two hospitals, US | Semi-structured interviews | thematic | To assess the motivations of PCa survivors for disclosing their diagnosis and treatment to close others, and their perceptions of their own others' responses to the disclosure |
| 1. Kelly, D. 2009. Changed men: The embodied impact of prostate cancer. Qualitative Health Research, 319(2), 151-163. 2. Kelly, D. 2004. Male sexuality in theory and practice. Nurs Clin North Am, 39, 341-56. | 14 | 0 | ns | (52-77) | ‘men of color' (21%) | Cancer Centre | Semi-structured interviews + Observation | Ethnography | (Paper 1) To examine masculine embodiment in the context of prostate cancer (Paper 2) To explore some of the more vulnerable aspects of male sexuality by considering the impact of PCa on the sexual dimension of men's lives |
| Kelly, D., Forbat, L., Marshall-Lucette, S., & White, I. 2015. Co-constructing sexual receovery after prostate cancer: A qualitative study with couples. Translational Andrology and Urology, 4(2), 131-8 | 10 | 8 | ns | Partners and men: (34-78) | Partners and men: White 56%, African Caribbean, Australian, Chinese, Greek, North African, Filipino, Taiwanese 44% | Hospitals | Semi-structured interviews + Observation | Thematic | To document the intimate experiences of men and their partners post-treatment, focussing particularly on qualitative accounts of the impact in relation to sexual functioning and how these concerns were managed between themselves, and discussed in the clinic |
| **Matsunaga D., Gotay C. 2004. Characteristics contributing to an enduring prostate Cancer Support Group in an Asian and Pacific Islander community. J Psychosoc Oncol, 22,4** | **24** | **n/a** | **mixed** | **(55 – 85)** | **17 (71%) were Asians or Pacific Islanders** | **East Hawaii Prostate Cancer Support Group, peer-run, in Hilo, on the island of Hawaii** | **Semi-structured interviews** | **Thematic content analysis** | **To provide a first look at an ongoing, well-established prostate cancer self-help group in a rural community in Hawaii. Contains statements around ethnicity but it is not clear who made them** |
| Sinfield, P., Baker, R., Ali, S. & Richardson, A. 2012. The needs of carers of men with prostate cancer and barriers and enablers to meeting them: a qualitative study in England. Eur J Cancer Care (Engl), 21, 527-34. | 0 | 33 | ns | (22–54) | White 73.5%, Black 14.7%, Asian 8.8%, Other 2.9% | Urology clinics, charities | Semi-structured interviews + Focus groups | Thematic; framework approach | To explore the needs of carers of men with PCa and to identify barriers and enablers to meeting these needs |
| Tanner, T., Galbraith, M. & Hays, L. 2011. From a woman's perspective: life as a partner of a prostate cancer survivor. J Midwifery Womens Health, 56, 154-60. | 0 | 113 | ns | M 65 | 87% were white | ns | Open ended questions on questionnaire (Questionnaires focused on health-related quality of life, health status, and relationship satisfaction) | Qualitative content analysis | To provide a better understanding of the experiences of partners of prostate cancer survivors |
| Waller, J. & Pattison, N. 2013. Men's experiences of regaining urinary continence following robotic-assisted laparoscopic prostatectomy (RALP) for localised prostate cancer: a qualitative phenomenological study. Journal of Clinical Nursing, 22, 368-78. | 7 | 0 | ns | 51-60 (n=1) 61-70 (n=5) 71-80 (n=1) | White 70% African 15% Chinese 15% | An acute tertiary care hospital trust | Semi-structured interviews | Phenomenology | To explore men's contextualised experiences of regaining continence post-RALP |
| Wu, L. M., Diefenbach, M. A., Gordon, W. A., Cantor, J. B. & Cherrier, M. M. 2013. Cognitive problems in patients on androgen deprivation therapy: a qualitative pilot study. Urologic Oncology, 31, 1533-8. | 11 | 0 | ns | M 59.45 (42-69) | White 81.8%, African-American 9.1%, Arab 9.1% | Community | Semi-structured interviews | Content analysis | A pilot study to describe patients' experiences of cognitive changes since starting ADT |
| **STUDIES EXCLUDED BECAUSE EXTRACTS ARE PREDOMINANTLY FROM DOMINANT WHITE PARTICIPANTS** | | | | | | | | | |
| Beck, A. M., Robinson, J. W. & Carlson, L. E. 2013. Sexual values as the key to maintaining satisfying sex after prostate cancer treatment: The physical pleasure-relational intimacy model of sexual motivation. Archives of Sexual Behavior, 42, 1637-1647. | 17 | 17 | M 3.5y (1–8) | M (Men) 64 (53–84); M (Partner) 57 (36–81) | Partners and men: White 86%, Asian 7%, Hispanic/ Latino 7% | Community via advertise-ments, presentations, local cancer centres, support groups, Canada | Semi-structured interviews | Grounded theory (Strauss & Corbin, 1998) | To generate a theory to explain how some couples are able to successfully maintain satisfying sexual intimacy, while other couples are not |
| Bell, K. & Kazanjian, A. 2011. PSA testing: Molecular technologies and men's experience of prostate cancer survivorship. Health, Risk & Society, 13, 183-198. | 7 | 0 | 0 m to 'long term survivors' | ns | at least 25% of men from non-white ethnic backgrounds (including South Asian, North Asian and Afro-Caribbean) | Prostate cancer support groups | Semi-structured interviews + Observation at support groups | Thematic (not explicit approach) | To delineate the meanings that the PSA test holds for PCa survivors, and the impact it has on men’s experience of cancer and survivorship; their lives after the completion of primary cancer treatment |
| Kazer, M. W., Harden, J., Burke, M., Sanda, M. G., Hardy, J. & Bailey, D. E. 2011. The experiences of unpartnered men with prostate cancer: a qualitative analysis. J Cancer Surviv, 5, 132-41. | 17 | 0 | >36 | M 63 (47-72) | White 82%, Black 17% | Recruited for a larger study | Semi-structured interviews | Thematic (not explicit approach) | To understand how men without a partner manage a prostate cancer illness |

** Study quality scores may be greater than those of the relevant separate articles due to reporting differences in the papers with overlap rather than dupilication of methodology and method sections.
