# Supplementary material for: Ethnicity and the prostate cancer experience: a qualitative metasynthesis
Source: Psychooncology. 2016 Aug 23;25(10):1147–56. doi: 10.1002/pon.4222 (PMC5096040; doi:10.1002/pon.4222)
Supplement: Supplementary file 4 — Supporting info item [file PON-25-1147-s004.docx]

Suppl file 4: PRISMA diagram for ethnicity metasynthesis.

21 BME papers (13 studies covering 11 minority ethnic groups)

Excluded:

**18** BME papers not reporting separate data for BME groups excluded

3 BME papers reporting mainly dominant white group extracts

Records identified through database searching

Cinahl (1448); IBSS (334); Medline (932); Psycinfo (487); WOS (621); ERIC (23)

(Total n = 3845 to 15^th^ December 2015)

Full-text articles excluded, with reasons
(**n = 291)**

Not PCa (8)

Mixed cancers (47)

Quantitative only (60)

No excerpts for 1^st^ order constructs (40)

Not about impact (45)

Screening, diagnosis, initial information seeking (37)

HCP only (6)

Not empirical (32)

Not English (6)

Meta-ethnography (3)

Qualitative media analyses (2)

Unavailable (1)

Pre 2000 (4)

Records excluded
(n =1460)

Full-text articles assessed for eligibility
(n =474)

**Articles included in main qualitative synthesis
(n = 184)**

Records screened
(n = 1934)

Records after duplicates removed
(n =1934)

Additional records identified through other sources
(n = 28)

**Ethnicity subanalysis**

**42** papers potentially eligible

## Identification

## Eligibility

## Included

## Screening
