# Supplementary material for: Ethnicity and the prostate cancer experience: a qualitative metasynthesis
Source: Psychooncology. 2016 Aug 23;25(10):1147–56. doi: 10.1002/pon.4222 (PMC5096040; doi:10.1002/pon.4222)
Supplement: Supplementary file 5 — Supporting info item [file PON-25-1147-s005.docx]

# **Suppl file 5: Summary of studies included in the ethnicity metasynthesis.**

| **Author and year** | **Men with Pca (n)** | **Partners (n)** | **Time since diagnosis in months (range)** | **Age in years; m=mean, md=median, SD= standard deviation, (range)** | **Ethnicity n (%)** | **Setting** | **Data collection** | **Data analysis** | **Aims (PCa = prostate cancer)** | **Study quality score** | **Study quality rating** |
| --- | --- | --- | --- | --- | --- | --- | --- | --- | --- | --- | --- |
| Abel 2002 [36] | 7 | 0 | ns | 61.8 (51-72) | African American 100% | Prostate cancer focus groups, US | Semi-structured interviews | Phenomenology | How do AA men perceive and describe their experiences of having PCa? | 18 | Good |
| Fergus 2002 [37] | 18 | 0 | 3.7y (1-8.5y) | 65 (57-75) | Anglo or European descent, 78% Afro-Canadian 22% | Prostate cancer focus groups, GBT and Black community newspapers, GPs, personal contacts, Canada | Semi-structured Interviews | Grounded theory using ‘meaning units’ | To understand the subjective experiences of men who have had (or had to consider having) their sexual capacity revoked or severely compromised, often suddenly, by having undergone treatments designed to rid them of their prostate cancer or impede its progression | 18 | Good |
| Gannon 2010 [38] | 7 | 0 | ns | M 63 (58-70) | British 57%, Irish 14), African 14%, Asian 14% | Urology Department of a teaching hospital in London, UK | Semi-structured interviews | Foucauldian Discourse Analysis | To investigate how men attempt to construct and reconstruct masculinity following RP for PCa | 13 | Fair |
| Jones 2007 [42] | 14 | 0 | ns | (51–830 | African American 100% | Patients in a prostate cancer centre in central Virginia (in the “Bible Belt” of the USA), and snowballing from these, US | Semi-structured interviews | Hermeneutic (Interpretive) Phenomenology | To examine the cultural beliefs and attitudes of African American PCa survivors regarding the use of complementary and alternative medicine (CAM) modalities | 13 | Fair |
| Jones 2011 [43] | 23 | 0 | ns | M 73 (66-80) | African-American 100% | Primary care clinics, community-based centres (churches, barbershops, diners) from a segment of the “Bible Belt” of the USA | Focus groups/group interviews (terms used interchangeably but group interviews seems more accurate) | Hermeneutic (Interpretive) Phenomenology | To explore cancer support and financial issues related to cancer care experienced by African-American men with PCa | 21 | good |
| Ka’opua study | | | | | | | | | | *Study score 20*** | *Study score Good* |
| Ka'opua 2007 [44] | 0 | 28 | >36; M 8.3y (6-15y) | M 72.6 (55-86) | White 28.6%, Japanese 28.6%, Chinese 21%, native Hawaiian 10.7%, Filipino 10.7% | Recruited from a survey investigating quality of life among long-term (five years or greater post-diagnosis) prostate cancer survivors and their wives, American Pacific Islands | Semi-structured interviews | Thematic analysis with constant comparison | To identify wives' challenges at the nexus of long--term PCa survivorship and aging, describe the function of spiritually based resources in coping, and describe common themes in adaptation among wives using spiritually based resources | 16 | Fair |
| Ka'opua 2005 [45] | 0 | 26 | >36 | M 73.82 (60-82) | Japanese 50%, native Hawaiian 12%, Chinese 27%, Filipino 12% | Recruited from a survey investigating quality of life among long-term (five years or greater post-diagnosis) prostate cancer survivors and their wives, American Pacific Islands | Semi-structured interviews | Thematic analysis with constant comparison | To explore the adaptive process to long term PCa survival in a cohort of elderly Asian or Pacific Islander wives | 18 | Good |
| Maliski study | | | | | | | | | | *Study score: 22* | *Study score:: Good* |
| Maliski 2008 [46] | 95 | 0 | ns | (50-70+) (54 Latino and 28 African American/Black were under 65) | African-American/Black 37%, Latino 63%.  Nearly all Latino (n=59/60) and few African American/ Black (n=2/35) were born outside the US | Longitudinal survey of participants in a state-funded programme providing free PCa treatment for uninsured low-income men, local veterans medical centre; advertisements newspaper, focus groups, US | Semi-structured interviews | Grounded theory (Strauss & Corbin, 1998) | To develop a descriptive model of processes used by low-income African-American/Black and Latino men to maintain masculine identity with PCa treatment-related symptoms | 19 | Good |
| Maliski 2010 [47] | 18 | 0 | ns | M 64.33 (52-81) | African-American/ Black 100% - subsample of 2008 participants | Longitudinal survey of participants in a state-funded programme providing free PCa treatment for uninsured low-income men, local veterans medical centre; advertisements newspaper, focus groups, US | Semi-structured interviews | Grounded theory (Strauss & Corbin, 1998) | To explore the meaning of PCa treatment-related symptoms among low-income African-American and Latino men | 22 | Good |
| Maliski 2012 [48] | 60 | 0 | ns | 50-65 | US Latino 100% (Mexican men, only 2 born in US) | Longitudinal survey of participants in a state-funded programme providing free PCa treatment for uninsured low-income men, local veterans medical centre; advertisements newspaper, focus groups, US | Semi-structured interviews | Grounded theory (Strauss & Corbin, 1998) | To understand the perception of spirituality in dealing with treatment-related side effects among low-income, uninsured Latino men treated for PCa | 17 | Fair |
| Williams 2014 [9] |  | 14 | <12 | M 55.1 (36-63) | Latina partners of Latino men with PCa 100% | Longitudinal survey of participants in a state-funded programme providing free PCa treatment for uninsured low-income men, US | Semi-structured interviews | Thematic with constant comparison | To describe the experiences of low-income Latinas longitudinally as their husbands recovered from RP for PCa, as well as to develop a framework for understanding the strategies they used when caring for their husband | 20 | Good |
| Mitschke 2009 [49] | 42 | 58 friends and family | <12: 16 (39.0%) 2-5y: 14 (34.1%) 6-10y: 6 (14.6%) >10y: 5 (12.2%) | For all 50-59 (n=6; 16.2%)  60-69 (n=13; 35.1%)  70-79 (n=18; 48.6%) | White 43%, Japanese 38%, native Hawaiian 9%, Filipino 5%, Chinese 5% | Community (postal survey), American Pacific Islands US | Open-ended questions in survey | Thematic | To explore how the family members and friends of PCa survivors coped with the illness experience, and the assumption of various roles within the family system during illness, across ethnic groups representative of Hawaii | 12 | Fair |
| Nanton 2011 [50] | 16 | 0 | 6m-10y (20y for recurrence 2y) | Md 72.5 (50-83) | First generation African-Caribbean immigrants 100% | Community, US | Semi-structured interviews | Thematic | Effect of ethnicity on PCa experience | 20 | Good |
| Rivers study | | | | | | | | | | | |
| Rivers 2011 [5**1**] | 12 | 12 | 1-5y | M_Men_ 59.75 (51-70) | African American 100% | Cancer centre, client network of a non-profit state-based organization, US | Semi-structured interviews | Thematic with constant comparison, and content analysis to select dominant themes | To qualitatively examine and describe the psychosocial issues related to the sexual functioning of AA couples surviving prostate cancer | 17 | Fair |
| Rivers 2012 [5**1a**] |  |  |  |  |  |  |  |  |  |  |  |
| Singh 2005 [5**2**] | 27 | 0 | ns | M 71 (58-82) | Caucasian 48.1%, Japanese 29.6%, Chinese 11.1%, Filipino 7.4%, Korean 3.7% | Population based survey, US | Semi-structured interviews | Thematic | To compare the perceptions, beliefs, ideas, and experiences that contribute to the decision of prostate cancer patients to use or not to use CAM | 14 | Fair |
| Hamilton study | | | | | | | | | | 16 | Fair |
| Song 2012 [5**3**] | 13 | 0 | 3-18 | M 67 (61-79) | African American 100% | Participants from a psycho-educational intervention NIH-funded study on Managing Uncertainty in Early Stage C Prostate Cancer Study, US | Semi-structured interviews | Grounded theory (Strauss & Corbin, 1998) (secondary data analysis of main study data) | To describe the role and meaning of spirituality among African American PCa survivors (and also breast cancer survivors, excluded from our synthesis) | 14 | Fair |
| Hamilton 2007 [39] | 13 | 0 | 3-18 | M 67 (61-79) | African American 100% | Participants from a psycho-educational intervention NIH-funded study on Managing Uncertainty in Early Stage C Prostate Cancer Study, US | Semi-structured interviews | Grounded theory (Strauss & Corbin, 1998) | To describe types of social support used by African American men to cope with experience of cancer | 16 | Fair |
| Hamilton 2004 [40] | 13 | 0 | 3-18 | M 67 (61-79) | African American 100% | Participants from a psycho-educational intervention NIH-funded study on Managing Uncertainty in Early Stage C Prostate Cancer Study, US | Semi-structured interviews | Grounded theory (Strauss & Corbin, 1998) | To explore the dynamics of supportive relationships from the perspective of older African Americans diagnosed with and treated for cancer. They are an exemplar population for studying reciprocal relationships | 15 | Fair |
| Hamilton 2003 [41] | 13 | 0 | 3-18 | M 67 (61-79) | African American 100% | Participants from a psycho-educational intervention NIH-funded study on Managing Uncertainty in Early Stage C Prostate Cancer Study, US | Semi-structured interviews | Grounded theory (Strauss & Corbin, 1998) | To better understand the cancer experiences of African American patients | 15 | Fair |
| Zhang 2014 [5**4**] | 74 | 0 | <36 | ns | African American 77%,  White 23% | Community, US | Semi-structured interviews | Thematic | To inquire about African-American cancer patients’ lived experiences of cancer and their attitudes toward cancer diagnosis and treatment, by comparing depressed and non-depressed African-American cancer patients and compared both groups with depressed White cancer patients, to identify attitudinal themes across groups and their relationship to long-term mental well-being | 18 | Good |
