# Supplementary material for: Ethnicity and the prostate cancer experience: a qualitative metasynthesis
Source: Psychooncology. 2016 Aug 23;25(10):1147–56. doi: 10.1002/pon.4222 (PMC5096040; doi:10.1002/pon.4222)
Supplement: Supplementary file 6 — Supporting info item [file PON-25-1147-s006.docx]

# **Suppl file 6: First second and third order constructs derived from the included studies, manifest effect sizes**

| First order | Second order | Third order | Manifest effect size | |
| --- | --- | --- | --- | --- |
|  | | | By article number | By study number |
| Spirituality and men’s coping [9,39,42-45,47-51,53,54,55,58] | The spiritual partnership or triumvirate alliance [9,39, 42-46,48,50-51]  Resignation to fate or God’s will [9,42,47-51,54,58] | **Spiritual alliances and the development of resilience and empowerment**  **[9,39,42-48,53-54,55,58]** | 71% | 69% |
| Spirituality and partner coping [9,47,48,51,54,55,58] | Positive reframing empowered by God’s strength [9,39,50,51,55] |  |  |  |
|  | Increasing and decreasing spirituality [39,42, 46,50, 51,54,55] |  |  |  |
| Cultural and ethnic-specific adversity as transformative and strengthening [9,47,48,58] | A lifelong fighting spirit and cultural role models [47,48,50,58] | **One more thing in the lifelong fight against adversity [9,47,48,50,52,58]** | 29% | 23% |
|  | Standing up to cancer versus feeling defeated [9,58] |  |  |  |
|  | Normalization of adversity [9,47,48,52,58] |  |  |  |
| Scepticism about CAM [45]  Dissatisfaction with conventional treatment,[45,53,56]  Taking control of treatment and looking for the best therapy [53,56] | Education and modernity versus the superstition of the traditional [45,48]  Purity versus medicalisation [45,56] | **Old and new discourses and the status of CAM [45,48,53,56]** | 19% | 31% |
| Cultural pressures and selective disclosure [47,49,50,54,58] | A mutual silence and its burden [47,54]  Encouraging the man to talk to others [47,55]  Maintaining a front [40, 49] | **Cultural pressures to maintain a social front that conceals** **[40,47,49,50,54,55,58]** | 33% | 49% |
|  | Brotherhood and silence [49,58] |  |  |  |
|  |  |  |  |  |
| Learning to accept the impact on sexual functioning [39-41,49,50,53,54,55] | Hope, gratitude for life, and the life-sex balance[9,40,47-51,54,55]  Normalising ED with reference to age [9,39,41,47,49,51-54]  Cessation of sex aids as a marker for survival [50,51,54] | **Cultural differences in male self-identity and the phenomenon of shifting masculinities**  **[9,39-41,47-55]** | 57% | 58% |
| Sense of inadequacy from erectile dysfunction (ED) [39-41,49,54] | Being a provider [49]  Being different but not less of a man [39, 40,41,47,49,53,54,55]  The magnified psychosexual impact [39, 40,41,49, 53, 54, 55] |  |  |  |
|  |  |  |  |  |
|  |  |  |  |  |
| Wanting to warn the unaware [9,42,50,52,53,55] | The temporality of cancer [9,51]  The temporality of life [9,41,51,54] | **Surviving for others and a legacy after death** **[9,39,41,42-44,47-54,55]** | 71% | 69% |
|  | The end of treatment and the start of survival [50,51,54] |  |  |  |
|  | Adding value to life and building social capital[9,39,42-44,47-54]  Faith, a reshaped future and a legacy [9,39,42-44,47-51,53,54]  Living for the family through healthier behaviours [9,47,48,50,51,53,54,55] |  |  |  |
| Confidence in HCPs or its lack [39,40,45,53,57]  Giving, receiving and understanding information [47,48,49,53,54,57]  HCPs involving partners or not [47,48,53,54,57]  Feeling supported and prepared by HCPs or not [47,48,53,54,57] | Better treatment outcomes from better HCP support [57]  Patient centred care [45,53,57] | **The relationship with healthcare providers**  **[39,40,45,47-46,53,54,57,58]** | 51% | 69% |
| Respect and empathy from HCPs or not [40,49,53,57,58] |  |  |  |  |
| Earning capacity and the cancer [53,58] |  | **A lack of economic capital [9,46,50,52,53,58]** | 29% | 41% |
| Financial and physical stresses of treatment costs and access to services [9,46,50,52] |  |  |  |  |
