# Supplementary material for: Ethnicity and the prostate cancer experience: a qualitative metasynthesis
Source: Psychooncology. 2016 Aug 23;25(10):1147–56. doi: 10.1002/pon.4222 (PMC5096040; doi:10.1002/pon.4222)
Supplement: Supplementary file 7 — Supporting info item [file PON-25-1147-s007.docx]

**Supplementary File 7: Constructs that were similar in the main synthesis and the BME subsynthesis.**

These constructs reflected the broad emotional impact of having cancer and the emotional and support resources men and their partners used to cope. Renegotiated bonds with those close to the man were sometimes weakened but mostly strengthened and valued. The constructs in-common also described reductions in social activities due to physical vulnerabilities caused by the disease and treatment side effects, and the way all the men eventually tackled and overcame or learned to control or live with the problems.

| **Emotion work [9,36,40,47-51,** **50,51,55,58]** | This covers men’s expressions of psychological impact of or low or depressed mood from the cancer and its consequences [58], as noted also by partners [9,47], and the emotion work done by men and partners to cope [47,48,50,51,58]. This construct covers such strategies as:   - partner downplaying and minimising [9] - considering the consequences of treatment to be a trade-off for life [40,47-51,50,51,55] - hopeful acceptance [49-51] - holistic approaches e.g. Taoism [40], Chi and meditation for the partner [47] - a positive attitude [47,49,50,58] - refocussing and distraction [9,49] - pragmatism [50,58]/fatalism as a belief in the inevitability of fate[58], the latter “a known belief in the African-American community[58] - normalising, particularly by partners [9,47], - downward comparison by men and partners [9, 58] and a positive world view of cancer[58] - partners’ use of patience and calm in the face of the man’s mood changes[9]   Much of the emotion work the men and their partners did was intended to protect the other, and this is far more evident in the literature around partners. Partners often disregarded and concealed their own feelings and needs in favour of the man’s. Some acted as selfless supporters[36], others through a sense of duty[9]. Men often suspected this emotion work [51]. Much emotion work by partners was to help the man cope with erectile dysfunction with women treading on eggshells or withholding affection to avoid upsetting the man[9,55] or to avoid his flareups[9,55], as well as the use of distractions[9], and putting up with new sexual positions to protect the man from his own feelings [9].  But emotion work often comes at a cost [9,47,55,68] and some women had feelings of guilt that they felt at all [55]. Authors of one paper noted that “Even though the women …. seemed mostly successful in their coping efforts …, their insistence that everything was normal in the face of the many other changes they described is dissonant[9]. |
| --- | --- |
| **The cancer journey [9,47,49,50]** | This metaphor is commonly used in relation to cancer. It involves coming to terms with the cancer and treatment side effects through a process of continuous learning and adaptation by both men and their partners [9,47,49,50]. There is a temporal element with some men staying in the present for longer than others as reported also in the main metasynthesis (see e.g. 97).For example Latino men believed that to embark on the journey (for example to try out erectile aids) would destroy their hopes of a return to full normality (including erectile function), whereas African American men were more pragmatic delay [50]. |
| **A new appreciation for a threatened life [45,46,49-52, 50-56,58]** | An emotional transformation caused by cancer as a ‘wake-up call’, with men re-evaluating their priorities in life. Often involving battle metaphors [50]. Distinct from the spiritual transformation described predominantly in BME papers. |
| **The importance of support from partners, family and social networks [9,36,42-44,46-50,52,55,]** | Partners were generally supportive, promoting men’s health e.g. through diet [46,47,52] providing emotional support, [46] practical support [46,52], acting as spokesperson to others [9] maintaining daily routines [46]. Examples are threaded through the main paper, intersecting with other constructs, though not all men benefited from this [46].  Social network members from friends and family and neighbours, to close community members through to weaker ties [44] all provided support for the man [43,44,46,50,52,55]. This gave the man a sense of stability [55], emotional support e.g. from just being there [43] or more actively [52], companionship support with distractions [43,52] or accompaniment to support groups [52], protecting and monitoring support [43], prayers [43], instrumental support [43,52], support for healthy behaviors [52], acting as spokesperson for the man and updating other family members or friends on the man’s condition [52], informational support (seeking out information or helping men to understand it) [43,52]  However some family members did not live near enough [52,55] or otherwise had poor relationships with the man [55], and one set of authors noted that some men did not want social support as they did not wanted to be treated as if an invalid [55].  Men gained significant benefits from talking to other men in their networks with prostate cancer including friends, family, local community groups and men met informally in hospital waiting rooms [55], as well as church members [55]. These were a source of informal support that contrasted with more formal support groups [36,42-44,49,50,52,55,]. The main benefit was exposure to tips and strategies on coping or managing the cancer and its treatment; advice on treatments by someone who had experienced them and learning that men could survive the cancer [55] were also important. Internet fora were a source of support with similar benefits [55].  Hamilton and Sandelowski [44] suggested this was centred around a reciprocating system of especial relevance to African Americans, that had been used for generations to survive racial oppression and economic hardships [44]. Certainly it was noted in two papers that African American/black men talked about a community brotherhood while Latino men did not [49,58].  Partners also obtained support from others [46-49,52,51,55], mainly from friends and family [55] including siblings, children and grandchildren [46]. This was mostly instrumental and emotional support [46], companionship and distraction [46].  Some family members were also healthcare professionals and so provided expert informational support [43]. One group of authors commented that the African Americans in their study preferred to obtain health-related information from knowledgeable family and friends rather than healthcare professionals and that this might be linked to ‘the history of institutional racism in the United States’ [43]. |
| **The impact on close relationship bonds [9,36,41,43,44,46-52,51,55,58]** | In the BME studies, and also in the main synthesis, men and partners often adopted a mutual silence to avoid straining their relationship [47, 48, 55]. There were some differences between the syntheses however. Two BME studies considering partners related this to concerns about offending cultural norms that prevented talk about intimate problems [47,48,51,55]. Men from the dominant ethnic groups would talk about the cancer and intimate problems at some point, to enable them to negotiate their relationship with their partner, whereas some men in the BME studies did not discuss the issues or their cancer at all, which one partner attributed to her Japanese husband’s ‘samurai streak’ [48].  However, this ‘cultural silence’ did not extend to all men [36, 55], for reasons that few papers explored. One suggested that the only significant difference in disclosure between BME and majority groups was in relaying the cancer diagnosis itself when the man was depressed, another said that conversations between partners regarding sexual dysfunction were the only ones to be unlikely in BME groups specifically [55]. Authors implicated a combination of a tendency to male self-reliance, concern for their partner driven by a fear of death, the stigma of the cancer, and also a lack of information from healthcare teams about strategies to overcome erectile dysfunction [51,55,58] all of which were also true for men in the main synthesis.  Moreover many included studies reported that the cancer strengthened couple’s relationships and that men valued the support of their partner, found also in the main metasynthesis. Thus overall the BME and main synthesis studies may be considered broadly similar with regard to this construct, as described further below.  Cancer was seen as affecting the whole family and not just the man [52]. This could:   - make relationships closer according to men [52] and partners [9], which can be linked to the greater value the men put on their partners [43,44,49-51,55] as they experienced their wake-up call (see theme above) [46,52] or enjoyed family support (see social support and partner support constructs) or shifted their ideas of masculinity (see main paper) [36,41,47-49,51,55]. - worsen relationships; often these were already not good quality [9] - leave relationships unchanged: - good relationships that were simply maintained; this was associated with less serious prognoses [52] - relations with geographically too distant friends and family [52] |
| **Embodied vulnerabilities and reduced social activities [36,41,49,50]** | Relates to men staying at home or otherwise reducing previous social activities and hobbies because of bodily problems such as incontinence, fatigue and pain caused by the cancer or its treatment. |
| **Taking control of the body and its future [49,50]** | Men tackling and overcoming or learning to control or live with the physical effects of the cancer and its treatment as an active process [50]. Some Latino men chose instead to wait passively for improvements [49]. Thus these men chose avoidance coping behaviours. They tended to relinquish control to God in so doing, which was not noted in the men in the main synthesis. See the masculinities construct in the main paper. |
